# The Study of Dynamic Modeling and Multivariable Feedback Control for Flexible Manipulators with Friction Effect and Terminal Load

**DOI:** 10.3390/s21041522

**Published:** 2021-02-22

**Authors:** Fuli Zhang, Zhaohui Yuan

**Affiliations:** School of Automation, Northwestern Polytechnical University, Xi’an 710072, China; yuanzhaohui2017@sina.com

**Keywords:** flexible robot, coupled dynamic model, coulomb friction, terminal load

## Abstract

The flexible manipulato is widely used in the aerospace industry and various other special fields. Control accuracy is affected by the flexibility, joint friction, and terminal load. Therefore, this paper establishes a robot dynamics model under the coupling effect of flexibility, friction, and terminal load, and analyzes and studies its control. First of all, taking the structure of the central rigid body, the flexible beam, and load as the research object, the dynamic model of a flexible manipulator with terminal load is established by using the hypothesis mode and the Lagrange method. Based on the balance principle of the force and moment, the friction under the influence of flexibility and load is recalculated, and the dynamic model of the manipulator is further improved. Secondly, the coupled dynamic system is decomposed and the controller is designed by the multivariable feedback controller. Finally, using MATLAB as the simulation platform, the feasibility of dynamic simulation is verified through simulation comparison. The results show that the vibration amplitude can be reduced with the increase of friction coefficient. As the load increases, the vibration can increase further. The trajectory tracking and vibration suppression of the manipulator are effective under the control method of multi-feedback moment calculation. The research is of great significance to the control of flexible robots under the influence of multiple factors.

## 1. Introduction

A flexible robot is characteristically lightweight, with high flexibility and a large load [1]. Through the structure design, the robot itself can be further optimized by considering the flexibility factor [2]. Since the robot design needs to meet the actual working conditions, designing its control method poses additional difficulties [3]. In practice, the stiffness change is absolute; that is, the elastic deformation is absolute. The dynamics of flexible robots are considered based on the comprehensive factors of deformation and control [4]. Therefore, dynamic modeling and characteristic analysis of flexible robots are essential.

Flexible manipulators have incomparable advantages over rigid manipulators, so current research is more inclined to focus on flexible manipulators [5]. But flexible manipulators have many limitations, including control complexity, uncertainty, MIMO and nonlinear systems, control overflow, and observed overflow [6]. The main reason for the complexity is the selection of dynamic models [7]. In the past three decades, people have developed many discrete methods for flexible robots. These methods include the finite element method (FEM), assumed mode method (AMM), and lumped parameter method [8]. Dynamics modeling methods are usually established based on AMM and Lagrangian methods. These methods have the advantages of high computational efficiency and flexible selection of boundary conditions [9]. Fukuda et al. established the dynamic characteristics of the two-link flexible manipulator under the influence of payload and gravity. He only analyzed and studied the effect of bending vibration on the manipulator [10]. Buffinton et al. [11] took the translation member of the standard arm as the elastic beam and established the motion equation containing the translation movement of the elastic member of the standard arm. Using the finite variable mode amplitude and space characteristic mode function, he further established the dynamic equation. Rahimi and Nazemizadeh [12] present a new method of selecting linkage boundary conditions and their modal eigenfunctions. They further used a dynamic model to discuss its deflection in terms of configuration vibration modes. Yi and Chen [13] presented dynamic modeling of the combined payload using assumed mode method (AMM). Moreover, he conducted numerical and experimental verification. Scaglioni and Ferretti [14] proposed a closed type dynamics model of a highly flexible robot based on Neutons-Euler. They used Matlab/Simulink, Modelica/Dymola, MSC/Adams and other software to verify the model. Khairudin et al. [15] built a two-link flexible robot to carry out the dynamic model based on comprehensively considering the influence of inertia and load on the damping hub of the structure, and they verified the effectiveness of the model through numerical simulation. Pradhan and Subudhi [16] used recursive extended Least squares (RELS) to achieve real-time identification of the NARMAX model. The model was verified by considering different loads and the experimental results were compared with the NARMAX model. Khairudin et al. [17] proposed a dynamic modeling and characterization of plane two-link flexible manipulator TLFM with payload. Using the Euler–Lagrange model and the AMM model, they established the dynamic model of the manipulator, which was verified by numerical simulation and experiment. Based on the above studies, the Lagrangian method was used to study the dynamic modeling of flexible robots from the perspective of its boundary conditions and load characteristics. In this paper, the boundary between the central rigid body and the load characteristics is considered comprehensively, and the dynamics are further constructed.

The energy source of robot movement mainly depends on the driving moment of joint, in which the energy loss of the robot must be considered. Many scholars mainly focus on the study of joint friction loss. At present, the most common friction models are the Stribeck friction model, the coulomb-Viscous friction model, the mane model, and the coulomb friction model [18]. Alinalbu et al. [19] used both coulomb friction and static friction models to describe the friction phenomenon of joint motion, and established a dynamic model considering both joint flexibility and friction. They analyzed the influence of friction on the dynamic characteristics of flexible joints. Moreno J et al. [20] took the two-degree-of-freedom manipulator as the research object and established the dynamic model of the viscous frictional manipulator. They further analyzed the influence of friction at the joints on the dynamic characteristics and end position errors of the manipulator. He et al. [21] used the LuGre friction model to establish the joint dynamics model containing friction, and analyzed the energy consumption caused by friction at robot joints. Wu et al. [22] proposed an extended joint friction model and analyzed the joint friction, finding that the low-speed motion performance of the mechanical arm deteriorates, and the operation accuracy is greatly affected. Taking a space manipulator with two degrees of freedom (2-DOF) as the research object, Liu et al. [23] solved the friction force based on the Coulomb friction model, and they explored and analyzed the influence of joint friction on the driving force of a space manipulator joint. The results show that gravity has a great influence on the dynamic characteristics of the manipulator. By integrating Coulomb’s friction theory with the Lagrangian multiplier algorithm, Dou and Yue [24] derived a friction model with sliding and viscous states. They analyzed the energy loss caused by friction in the collision system. Therefore, the coulomb friction model is used to solve the friction moment at the joint of the manipulator. The force of friction is proportional to the normal load. It is opposite to the direction of relative motion and does not depend on the friction theory of contact area. This coulomb friction model is relatively intuitive and easy to calculate and apply [25].

Many control methods of flexible robots have been developed and improved based on classical control methods of traditional rigid robots. The proportional differential/proportional differential integral (PD/PID) controller with position feedback and state feedback control with tracking and calculating torque was modified for flexible manipulator operation. Moreover, further control studies for different payloads were conducted. Mohamed et al. [26] used a linear matrix inequality PD (LMI PD) controller to control the position and end vibration suppression of the two-link flexible manipulator under the condition of changing the load effect. The robustness of the control technology is verified by the comparison between numerical simulation and experimental results. Walsh et al. [27] proposed the modeling and control of a scalable, flexible robot. The terminal position control of manipulator using a PD controller was realized. Matsuno and Yamamoto [28] used singular perturbation technology to divide the dynamic system into a slow subsystem and a fast subsystem. They further adopted state feedback control to control and suppress vibration at the end of the flexible two-link. Sawada and Itamiya [29] used feedback linearized extension (calculated torque method) to control the position of the flexible robot. In the case of uncertainty and interference, the proposed method was numerically simulated. Vandini et al. [30] proposed a flexible two-link motion control based on a visual sensor in which feedback signals are generated by visual sensors and PID controllers are used to control the movement of flexible robots. Chu and Cui [31] propose a method to actively suppress the internal vibration of the two-link flexible manipulator to adapt to the variation of model parameters. The model parameters are composed of input shaper and multi-mode adaptive positive-position feedback.

In this paper, coupling characteristics between joint friction, terminal load, and flexible machinery are considered comprehensively. Taking the structure of the central rigid body-rotating flexible Euler–Bernouli beam-terminal load as the research object, the dynamics of the flexible manipulator are established by using assumed modes method (AMM) and the Euler-Lagrange method. Finally, the dynamics of the flexible manipulator are verified and its characteristics are analyzed by simulation. The vibration characteristics of the manipulator under different loads and friction conditions are analyzed respectively. The coupling dynamics of the flexible robot are further decomposed and the track tracking and vibration suppression are carried out by using multiple feedback control methods.

## 2. Dynamic Modeling of the Flexible Robot

**Figure 1:**
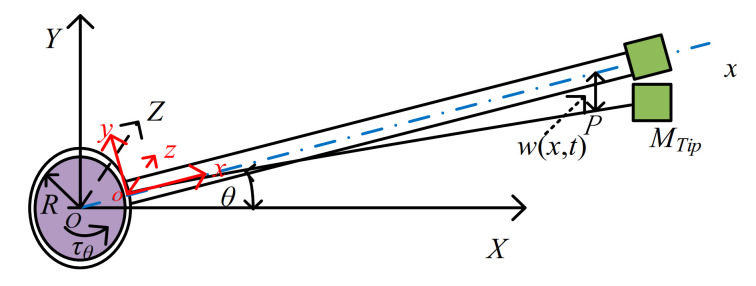
The deformation diagram of the central rigid body rotating flexible beam.

The structure of the flexible robot is mainly composed of motors, long links, terminal grasping mechanisms, and loads. The concrete structure is simplified as a central rigid body, flexible manipulator, and terminal load. The structure is shown in Figure 1.

In Figure 1, the manipulator structure motor drives the rotor, profile, flexible beam, and end load. The drive motor shaft is located at the fixed end with a rotor radius *R*. This coordinate system is the inertial coordinate system *O*-*XYZ*. The connection point between the contour and the beam is the following coordinate system is *o*-*xyz*. And the rotation between the two frames is *θ*, so it is a rigid rotation. Its rotational moment is *τ_θ_*. The length of the manipulator is *L*, and the density of the mechanical manipulator *ρ*. The load mass of the free end is *M_L_*. The position and velocity of any point *p* on the flexible robot and the position and velocity of the end load are shown in Equation (1).

(1) $$\left\{\begin{array}{l} r=\left[\begin{array}{c} (R+x)cos\theta -w(x,t)sin\theta \\ (R+x)sin\theta +w(x,t)cos\theta \end{array}\right]\\ \dot{r}=\left[\begin{array}{c} -\dot{w}(x,t)sin\theta -\left(\left(R+x\right)sin\theta +\dot{w}(x,t)cos\theta \right)\dot{\theta }\\ \dot{w}(x,t)\cos\theta +\left(\left(R+x\right)cos\theta -\dot{w}(x,t)\sin\theta \right)\dot{\theta } \end{array}\right] \end{array}\right.$$

Its length *L* is substituted into Equation (1), and the terminal position and velocity of the flexible manipulator are obtained.

The position vector of flexible deformation of point *p* on the flexible manipulator is *w*(*x*, *t*). The *N*-dimensional modal functions are selected by the modal truncation method. The displacement vector of the flexible deformation is expressed as Equation (2).

(2) $$w(x,t)=\sum_{i=1}^{N}\phi_{i}\left(x\right)\varepsilon \left(t\right)$$

where *ϕ_i_*(*x*) is the modal function, *ε_i_*(*t*) is the generalized coordinates.

According to the existing research on the cantilever beam, the deformation of flexible manipulator was analyzed. Based on the flexural elasticity of material mechanics, when *L*/*hz* > 10, *L*/*h_b_* > 10, the flexible manipulator is described by the Euler–Bernoulli beam. The axial deformation and shear deformation were not taken into account. The parameters *h_z_* and *h_b_* were the height and width of the flexible manipulator.

According to the bending vibration principle, the forced bending equation of the flexible manipulator is expressed as Equation (3).

(3) $$EI\frac{\partial^{4}w\left(x,t\right)}{\partial x^{4}}+\rho S\frac{\partial^{2}w\left(x,t\right)}{\partial t^{2}}=F\left(x,t\right)$$

where *E* is Modulus of elasticity, *I* is Moment of inertia section, *S* is Cross-sectional area, *F*(*x*, *t*) is the external force.

To solve its general solution, the free vibration Equation (4) is obtained.

(4) $$EI\frac{\partial^{4}w\left(x,t\right)}{\partial x^{4}}+\rho A\frac{\partial^{2}w\left(x,t\right)}{\partial t^{2}}=0$$

Using the separate variables method, the bending variables are decomposed spatially and temporally.

(5) $$\frac{EI}{\phi \left(x\right)}\frac{\partial^{4}\phi \left(x\right)}{\partial x^{4}}=\frac{\rho A}{\tau \left(t\right)}\frac{\partial^{2}\tau \left(t\right)}{\partial t^{2}}$$

Equation (5) can be equivalent to a constant χ2. The equation is decomposed into Equations (6) and (7).

(6) $$\frac{\partial^{4}\phi \left(x\right)}{\partial x^{4}}-\beta \chi^{2}\phi \left(x\right)=0$$

(7) $$\frac{\partial^{2}\tau \left(t\right)}{\partial t^{2}}-\chi^{2}\tau \left(t\right)=0$$

where *β* is *EI*/*ρS*. Equation (6) is the variable function of the flexible manipulator space. Equation (7) is the time function of the flexible manipulator.

The solution of Equation (6) requires four boundary conditions. Firstly, four boundary conditions are determined by mechanical analysis of the bar. As the end point of the beam is fixed, the bending displacement and slope of the fixed end of the flexible manipulator are 0.

(8) $${\left.w\left(x,t\right)\right|}_{x=0}=0$$

(9) $${\left.\frac{\partial w\left(x,t\right)}{\partial x}\right|}_{x=0}=0$$

At the free end point, the bending moment and shear force of the beam are obtained under load.

(10) $${\left.\frac{\partial^{2}w\left(x,t\right)}{\partial x^{2}}\right|}_{x=L}={\left.\frac{\partial^{2}\phi \left(x\right)}{\partial x^{2}}\right|}_{x=L}=0$$

(11) $${\left.\frac{\partial^{3}w\left(x,t\right)}{\partial x^{3}}\right|}_{x=L}={\left.\frac{\partial^{3}\phi \left(x\right)}{\partial x^{3}}\right|}_{x=L}=M_{L}\frac{\partial^{2}w\left(L,t\right)}{\partial t^{2}}$$

where *M_L_* is the mass of the end load. Equation (11) is the shear force under load, and the right formula of Equation (11) is the product of the acceleration and mass at the end of the link under bending changes.

The parameter *βχ*^2^ is equivalent to *α*^4^.

(12) $$\frac{\partial^{4}\phi \left(x\right)}{\partial x^{4}}-\alpha^{2}\phi \left(x\right)=0$$

The general solution of Equation (6) is Equation (13).

(13) $$\phi \left(x\right)=C_{1}\cos\alpha x+C_{2}\sin\alpha x+C_{3}\cosh\alpha x+C_{4}\sinh\alpha x$$

In the general solution formula, *C*_1_, *C*_2_, *C*_3,_ and *C*_4_ can be solved by the above equations.

(14) $$\partial \phi \left(x\right)/\partial x=\alpha \left(-C_{1}\sin\alpha x+C_{2}\cos\alpha x+C_{3}\sinh\alpha x+C_{4}\cosh\alpha x\right)$$

(15) $$\partial^{2}\phi \left(x\right)/\partial x^{2}=\alpha^{2}\left(-C_{1}\cos\alpha x-C_{2}\sin\alpha x+C_{3}\cosh\alpha x+C_{4}\sinh\alpha x\right)$$

(16) $$\partial^{3}\phi \left(x\right)/\partial x^{3}=\alpha^{3}\left(C_{1}\sin\alpha x-C_{2}\cos\alpha x+C_{3}\sinh\alpha x+C_{4}\cosh\alpha x\right)$$

Substituting Equations (8)–(11) into Equations (14)–(16), the frequency equation and mode function of the single flexible manipulator can be obtained.

(17) $$\phi_{i}\left(x\right)=\left(\cosh\left(\alpha_{i}x\right)-\cos\left(\alpha_{i}x\right)\right)-\frac{\cosh\left(\alpha_{i}L\right)+\cos\left(\alpha_{i}L\right)}{\sinh\left(\alpha_{i}L\right)+\sin\left(\alpha_{i}L\right)}\left(\sinh\left(\alpha_{i}x\right)-\sin\left(\alpha_{i}x\right)\right)$$

Using the above boundary conditions, the vibration frequency equation can be obtained.

(18) $$1+\cos\left(\alpha_{i}l\right)\cosh\left(\alpha_{i}l\right)=\left(M_{L}/\rho SL\right)\left(\alpha_{i}l\right)\left(\sin\left(\alpha_{i}l\right)\cosh\left(\alpha_{i}l\right)-\cos\left(\alpha_{i}l\right)\sinh\left(\alpha_{i}l\right)\right)$$

where *α_i_*(*i* = l, 2, …) is the eigenvalue of the system, and its corresponding natural frequency of vibration is Equation (19).

(19) $$\beta ={\left(\alpha l\right)}^{2}\sqrt{EI/\rho l^{4}}$$

By Equations (18) and (19), each vibration natural frequency *β* corresponds to a particular vibration mode function *ϕ*(*x*) and time function coefficient tau *τ*(*t*). By using the principle of linear superposition, the solution of Equation (20) can be obtained.

(20) $$w\left(x,t\right)=\sum_{i=1}^{\infty }\phi \left(x\right)\tau \left(t\right)$$

The kinetic energy of the flexible manipulator is obtained by using the Lagrange method.

(21) $$\begin{array}{l} T=\frac{1}{2}J{\dot{\theta }}^{2}+\frac{1}{2}\bar{m}\sum_{i=1}^{N}\int_{0}^{L}\phi_{i}^{2}\left(x\right){\left(\frac{\tau_{i}\left(t\right)}{dt}\right)}^{2}dx+\frac{1}{2}\bar{m}\dot{\theta }\sum_{i=1}^{N}\int_{0}^{L}\phi_{i}^{2}\left(x\right){\left(\frac{\tau_{i}\left(t\right)}{dt}\right)}^{2}dx+\\ \;\;\;\;\;\bar{m}\sum_{i=1}^{N}\int_{0}^{L}\left(R+x\right)\phi_{i}^{2}\left(x\right){\left(\frac{\tau_{i}\left(t\right)}{dt}\right)}^{2}dx+m_{p}\left({\left(L\dot{\theta }\right)}^{2}+\left(1+{\dot{\theta }}^{2}+2L\dot{\theta }\right)\sum_{i=1}^{N}{\left(\phi_{i}\left(L\right)\tau_{i}\left(t\right)\right)}^{2}\right) \end{array}$$

where $\bar{m}=M/l$ is mass per unit length.

The potential energy of the flexible manipulator is obtained.

(22) $$V={\sum_{i=1}^{n}\int_{0}^{L}EI\left(\frac{\partial^{2}\phi_{i}\left(x\right)}{\partial x^{2}}\right)}^{2}q_{i}^{2}dx+\frac{1}{2}mgL\cos\theta +m_{p}gL\cos\theta$$

In Equation (22), the first term is the energy generated by the deformation of the flexible manipulator. The second and third items are the gravitational potential energy of the flexible manipulator and the end-point load respectively. According to the Lagrange equation of the second kind, the dynamic equation of the flexible manipulator can be obtained.

(23) $$\left\{\begin{array}{l} L=T-V\\ \frac{d}{dt}\left(\frac{\partial L}{\partial {\dot{q}}_{i}}\right)+\frac{\partial L}{\partial q_{i}}=Q_{i} \end{array}\right.$$

where *q_i_* = [*θ*, *q*_1_, *q*_2_,……*q_n_*]*^T^*is the generalized coordinates of flexible manipulator,*Q*_i_ = [*τ_θ_*_,_0,….0]*^T^*is the generalized force of the flexible manipulator.

The dynamics of a flexible manipulator is expressed as an equation of state.

(24) $$\left[\begin{array}{cc} m_{11} & m_{12}\\ m_{21} & m_{22} \end{array}\right]\left[\begin{array}{l} \ddot{\theta }\\ {\ddot{q}}_{1}\\ \vdots \\ {\ddot{q}}_{n} \end{array}\right]+\left[\begin{array}{l} C_{0}\left(\theta ,\theta ,q,\dot{q}\right)\\ C_{1}\left(\theta ,\theta ,q,\dot{q}\right)\\ \vdots \\ C_{N}\left(\theta ,\theta ,q,\dot{q}\right) \end{array}\right]+\left[\begin{array}{cccc} 0 & & & \\ & K_{1}q_{1} & & \\ & & \ddots & \\ & & & K_{N}q_{N} \end{array}\right]+\left[\begin{array}{l} G\\ 0\\ \vdots \\ 0 \end{array}\right]=Q$$

where

(25) $$\left\{\begin{array}{l} \begin{array}{l} m_{11}=J+m_{L}\left(L^{2}+\sum_{i=1}^{N}\phi_{i}^{2}\left(L\right)q_{i}{}^{2}\right)+\bar{m}\sum_{i=1}^{N}\int_{0}^{L}\phi_{i}^{2}\left(x\right)q_{i}{}^{2}dx\\ m_{12}=m_{21}{}^{T}=[\gamma_{1},\gamma_{2,}\ldots \gamma_{N}]\\ \gamma_{n}=\bar{m}\int_{0}^{L}\left(R+x\right)\phi_{i}\left(x\right)dx+M_{L}L\phi_{i}\left(L\right)\begin{array}{cc} & n=\left(1,2\ldots N\right) \end{array} \end{array}\\ \begin{array}{l} m_{22}=diag\left(\left[m_{1},m_{2},\ldots ,m_{N}\right]\right)\\ m_{i}=\bar{m}\int_{0}^{L}\phi_{i}^{2}\left(x\right)dx+m\phi_{i}^{2}\left(L\right) \end{array} \end{array}\right.$$

(26) $$\left\{\begin{array}{l} C_{0}=2\dot{\theta }\sum_{n=1}^{N}\bar{m}{\dot{q}}_{n}q_{n}\int_{0}^{L}\phi_{n}^{2}\left(x\right)dx+2\dot{\theta }\sum_{n=1}^{N}m{\dot{q}}_{n}q_{n}\phi_{n}^{2}\left(L\right)\\ C_{n}=-\bar{m}\int_{0}^{L}\phi_{n}^{2}\left(x\right){\dot{\theta }}^{2}q_{n}dx+m\phi_{n}^{2}\left(L\right){\dot{\theta }}^{2}q_{n}\\ K_{n}=EI{\int_{0}^{L}\left(\frac{d^{2}\phi_{i}\left(x\right)}{dx^{2}}\right)}^{2}dxq_{n}\\ G=\frac{1}{2}mgL\cos\theta +m_{p}gL\cos\theta \\ Q=\left[\begin{array}{cccc} \tau & 0 & \cdots & 0 \end{array}\right] \end{array}\right.$$

where the state matrix of *m_ij_* (*i*, *j* = 1, 2) is the mass inertia matrix, which is symmetric and bounded. *C* is the coupling matrix of Coriolis force and centrifugal force. *K* is the stiffness matrix, *G* is the gravity matrix,*Q* is the matrix of the input torque. The specific equation of state implementation is given in the Appendix A.

## 3. Improved Friction Model

**Figure 2:**
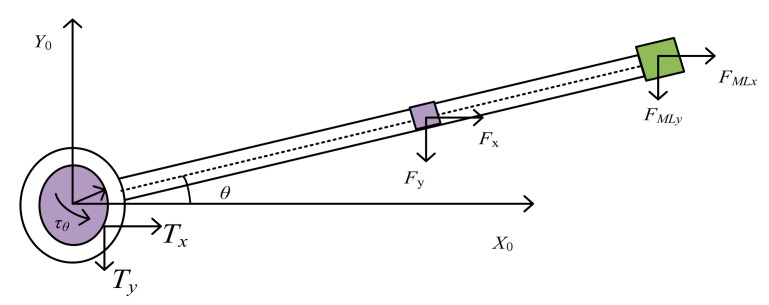
The friction model of flexible manipulator.

Based on the analysis of the instantaneous static equilibrium principle, the friction torque of articulation at the joint is obtained based on the Coulomb friction force. It is shown in Figure 2. The improved friction model takes into account the influence of the flexible characteristics of the manipulator and the lumped mass of the end. Therefore, the friction torque at the joint is recalculated [20].

According to the velocity vector in Equation (1), the acceleration vector at any point on the manipulator can be obtained through further derivation.

(27) $$\left\{\begin{array}{ll} \ddot{x}= & -\left(\left(R+x\right)\sin\theta +\dot{w}\left(x,t\right)\cos\theta \right)\ddot{\theta }-\left(\left(R+x\right)\sin\theta +\dot{w}\left(x,t\right)\cos\theta \right){\dot{\theta }}^{2}-\\ & \left(\dot{w}\left(x,t\right)\cos\theta +\ddot{w}\left(x,t\right)\cos\theta \right)\dot{\theta }-\ddot{w}\sin\theta \\ \ddot{y}= & -\left(\left(R+x\right)\cos\theta +\dot{w}\left(x,t\right)\sin\theta \right)\ddot{\theta }-\left(\left(R+x\right)\sin\theta +\dot{w}\left(x,t\right)\cos\theta \right){\dot{\theta }}^{2}\\ & -\left(\dot{w}\left(x,t\right)\cos\theta +\ddot{w}\left(x,t\right)\cos\theta \right)\dot{\theta }+\ddot{w}\cos\theta \end{array}\right.$$

As *x* = *L* is substituted into Equation (27), the acceleration vector of the terminal load $\left[\begin{array}{cc} \ddot{x}\left(L\right) & \ddot{y}\left(L\right) \end{array}\right]$ is obtained. The force at any point on the mechanical arm is decomposed and projected on the *XOY* plane, and *F_x_* and *F_y_* can be obtained. Since the flexible manipulator is studied by distributed parameters, the projection force can be obtained by using the fixed-base integral method and the force balance principle.

According to D’alembert’s principle and Newton’s second law, the forces on each microunit of the manipulator are obtained. The force is integrated over the length range of the manipulator arm [0, *L*] and the force components on the mechanical arm members in *x* and *y* directions are obtained.

(28) $$F_{x}=\rho_{1}S_{1}\int_{R}^{L}\ddot{x}dx,F_{y}=\rho_{1}S_{1}\int_{R}^{L}\ddot{y}dx$$

The force on the terminal load can be further calculated.

(29) $$F_{M_{L}x}=M_{L}\ddot{x}\left(L\right),F_{M_{L}y}=M_{L}\ddot{y}\left(L\right)$$

The contact forces for the joints of the flexible robot are obtained.

(30) $$\left\{\begin{array}{l} T_{x}=\rho_{1}S_{1}\int_{0}^{L}\ddot{x}dx+M_{L}\ddot{x}\left(L\right)\\ T_{y}=\rho_{1}S_{1}\int_{0}^{L}\ddot{y}dx+M_{L}\ddot{y}\left(L\right) \end{array}\right.$$

According to the Coulomb friction model, the friction moment of the joint is obtained.

(31) $$\tau_{f}=\tau_{fx}+\tau_{fy}=\mu T_{x}Rsgn\left(\dot{\theta }\right)+\mu T_{y}Rsgn\left(\dot{\theta }\right)$$

where *μ* is the friction coefficient.

The friction factor takes into account the effect of the flexibility characteristics on the mass of the end load. Combining Equations (24) and (31), a dynamic model with flexibility characteristics and terminal load is established.

(32) $$\left[\begin{array}{cc} m_{11} & m_{12}\\ m_{21} & m_{22} \end{array}\right]\left[\begin{array}{l} \ddot{\theta }\\ {\ddot{q}}_{1}\\ \vdots \\ {\ddot{q}}_{n} \end{array}\right]+\left[\begin{array}{l} C_{0}\left(\theta ,\theta ,q,\dot{q}\right)\\ C_{1}\left(\theta ,\theta ,q,\dot{q}\right)\\ \vdots \\ C_{N}\left(\theta ,\theta ,q,\dot{q}\right) \end{array}\right]+\left[\begin{array}{cccc} 0 & & & \\ & K_{1}q_{1} & & \\ & & \ddots & \\ & & & K_{N}q_{N} \end{array}\right]+\left[\begin{array}{l} G\\ 0\\ \vdots \\ 0 \end{array}\right]+\left[\begin{array}{l} \tau_{f}\\ 0\\ \vdots \\ 0 \end{array}\right]=Q$$

For convenience, Equation (32) is shortened to Equation (33).

(33) $$\left\{\begin{array}{l} M\left[\begin{array}{c} \ddot{\theta }\\ \ddot{q} \end{array}\right]+C\left(\theta ,\theta ,q,\dot{q}\right)+Kq+G+\tau_{f}=Q\\ \tau_{f}=\mu T_{x}Rsgn\left(\dot{\theta }\right)+\mu T_{y}Rsgn\left(\dot{\theta }\right) \end{array}\right.$$

## 4. The Controller Design for Multiple Closed-Loop Adaptive Computing Torques

**Figure 3:**
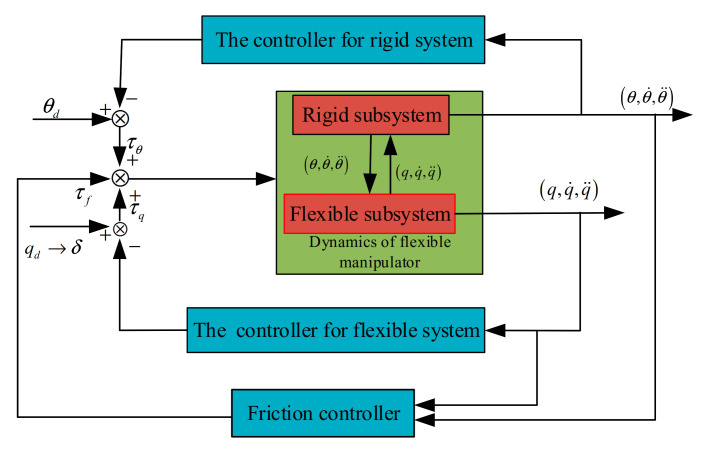
Design of flexible manipulator controller with multiple closed loops.

To better control terminal load and the coupling dynamics of flexible and friction characteristics, the dynamics of the flexible manipulator are first decomposed and multiple closed-loop controllers are applied to the manipulator controller. Secondly, considering that the two subsystems as the load at the end of the flexible deformation of its kinetic energy is smaller, we are therefore able to ignore the flexible deformation of the load in the end as well as the influence of the friction torque at the same time. The influence of the flexible and terminal load specific controller design is shown in Figure 3.

In Figure 3, *θ_d_* is the rigid joint trajectory, and *q_d_* is the specified trajectory of the flexible vibration. However, the flexible trajectory is a virtual quantity. According to the need of the vibration suppression target, the specified trajectory should be as small as possible or guaranteed to be within a small range. According to Equation (32), rigid motion and flexible vibration are interactive. The dynamics module of the flexible manipulator is represented. Based on the rigid feedback of the manipulator, the flexible feedback is designed to track the trajectory of the whole manipulator system. Compared with the previous singular perturbation method and intelligent method, this method is realized by decomposing the subsystem of the coupling system. The difference between them is that the flexible system constrains the control error by the virtual control objective, and it is equivalent in the way of the black box. The realization of the control method can also be identified by the intelligent control, but this will increase the amount of control tasks. According to Equations (27)–(31), both rigid and flexible variables are taken into account for joint friction. Therefore, rigid variables and flexible variables are introduced into the friction compensation design.

As the systems *M*, *C*, *G*, and *K* are deterministic systems, and they are known, Equation (33) is further derived.

(34) $$\left\{\begin{array}{l} m_{11}\ddot{\theta }+m_{12}\ddot{q}+C_{11}\dot{\theta }+C_{12}\dot{q}+G\left(\theta \right)+\tau_{f}\left(\dot{\theta },\dot{q}\right)=\tau \\ m_{21}\ddot{\theta }+m_{22}\ddot{q}+C_{21}\dot{\theta }+C_{22}\dot{q}+Kq=0\\ \tau_{f}=\mu T_{x}Rsgn\left(\dot{\theta }\right)+\mu T_{y}Rsgn\left(\dot{\theta }\right) \end{array}\right.$$

The second term in Equation (34) is substituted into the first term, and Equation (34) is further rewritten into Equation (35).

(35) $$M_{A}\ddot{\theta }+C_{A}\dot{\theta }+G\left(q\right)+M_{B}\ddot{q}+C_{B}\dot{q}+Kq+\tau \left(\dot{\theta },\dot{q}\right)=\tau$$

where $M_{A}=m_{11}+m_{21}$, $M_{B}=m_{12}+m_{22}$, $C_{A}=C_{11}+C_{21}$, $C_{B}=C_{12}+C_{22}$ As the expected trajectories ${\left[\theta_{d},{\dot{\theta }}_{d},{\ddot{\theta }}_{d}\right]}^{T}$ and ${\left[q_{d},{\dot{q}}_{d},{\ddot{q}}_{d}\right]}^{T}$ are given, the flexible vibration feedback control is carried out based on rigid motion. Equation (35) will be expressed as the dynamic equation of the error space.

(36) $$M_{A}\left(\ddot{\theta }-{\ddot{\theta }}_{d}\right)+C_{A}\left(\dot{\theta }-{\dot{\theta }}_{d}\right)+M_{B}\left(\ddot{q}-{\ddot{q}}_{d}\right)+C_{B}\left(\dot{q}-{\dot{q}}_{d}\right)+Kq+G\left(\theta \right)+\tau_{f}\left(\dot{\theta },\dot{q}\right)=\tau$$

Let $\underset{t\to \infty }{\lim}\left(\varepsilon_{d},{\dot{\varepsilon }}_{d},{\ddot{\varepsilon }}_{d}\right)$ tends to $\left(\delta ,\dot{\delta },\ddot{\delta }\right)$, δ,$\dot{\delta }$ and $\ddot{\delta }$ is minimal or 0.

(37) $$M_{A}\left(\ddot{\theta }-{\ddot{\theta }}_{d}\right)+C_{A}\left(\dot{\theta }-{\dot{\theta }}_{d}\right)+M_{B}\left(\ddot{\varepsilon }-\ddot{\delta }\right)+C_{B}\left(\dot{\varepsilon }-\dot{\delta }\right)+Kq+G\left(\theta \right)+\tau_{f}\left(\dot{\theta },\dot{q}\right)=\tau$$

According to the motion relation of unit mass system, the control closed-loop system satisfies the following conditions.

(38) $$\left\{\begin{array}{l} \ddot{\theta }={\ddot{\theta }}_{d}-k_{v}\left(\dot{\theta }-{\dot{\theta }}_{d}\right)-k_{p}\left(\theta -\theta_{d}\right)\\ \ddot{q}=\ddot{\delta }-k_{qv}\left(\dot{q}-\dot{\delta }\right)-k_{qp}\left(q-\delta \right) \end{array}\right.$$

Substituting Equation (37) into Equation (39), the joint driving torque is obtained.

(39) $$\begin{array}{l} \tau =M_{A}{\ddot{\theta }}_{d}+\left(C_{A}-M_{A}k_{v}\right)\left(\dot{\theta }-{\dot{\theta }}_{d}\right)-k_{p}\left(\theta -\theta_{d}\right)+\\ M_{B}\ddot{\delta }+\left(C_{B}-M_{B}k_{qv}\right)\left(\dot{q}-\dot{\delta }\right)-k_{qp}\left(q-\delta \right)+G\left(\theta \right)+\tau_{f}\left(\dot{\theta },\dot{q}\right) \end{array}$$

The control law is expressed in Equation (40).

(40) $$\tau =\hat{\tau }+\tau_{eq}+K_{q}q+G\left(\theta \right)+\tau_{f}\left(\dot{\theta },\dot{q}\right).$$

where $\hat{\tau }=M_{A}{\ddot{\theta }}_{d}+\left(C_{A}-M_{A}k_{v}\right)\left(\dot{\theta }-{\dot{\theta }}_{d}\right)-k_{p}\left(\theta -\theta_{d}\right)$ $\tau_{eq}=M_{B}{\ddot{q}}_{d}+\left(C_{B}-M_{B}k_{qv}\right)\left(\dot{q}-\dot{\delta }\right)-k_{qp}\left(q-\delta \right)$

According to the motion equation of the error space above, it can be seen that appropriate *k_v_*, *k_p_*, *k_qv_*, and *k_qp_* are selected. Substituting Equation (40) into Equation (34), Equation (41) can be obtained.

(41) $$\left(\ddot{\theta }-{\ddot{\theta }}_{d}\right)-k_{v}\left(\dot{\theta }-{\dot{\theta }}_{d}\right)-k_{p}\left(\theta -\theta_{d}\right)-k_{qv}\left(\dot{q}-\dot{\delta }\right)-k_{qp}\left(q-\delta \right)=0$$

Finally, the stability of the control system is proved.

## 5. Results

### 5.1. Characteristic Analysis of Friction and Terminal Load of the Flexible Manipulator

To verify the feasibility of the coupling dynamics, a series of simulations were carried out on a single degree of freedom flexible robot. The radius of the central rigid body is *R* = 0.02. The parameters of the manipulator are shown in Table 1. 

**Figure 4:**
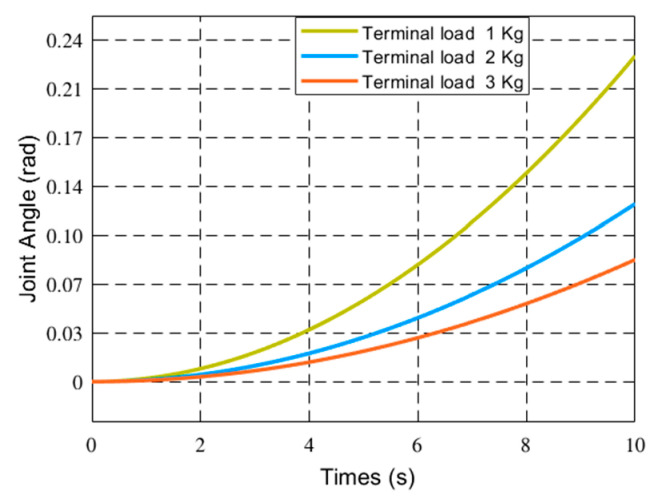
Joint Angle changes of the manipulator under different loads.

**Figure 5:**
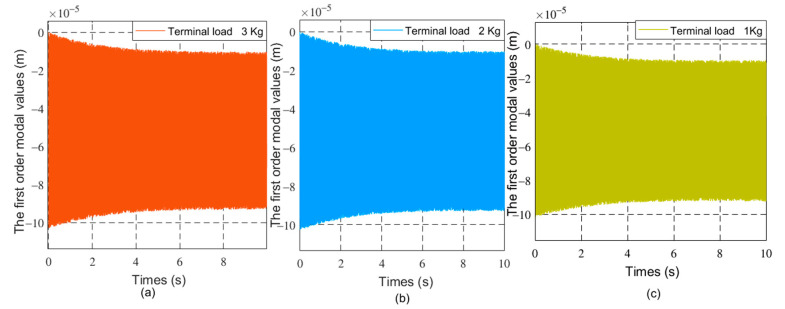
The first order modal changes of the manipulator under different loads, (**a**) 30 kg, (**b**) 20 Kg, (**c**) 10 Kg.

**Figure 6:**
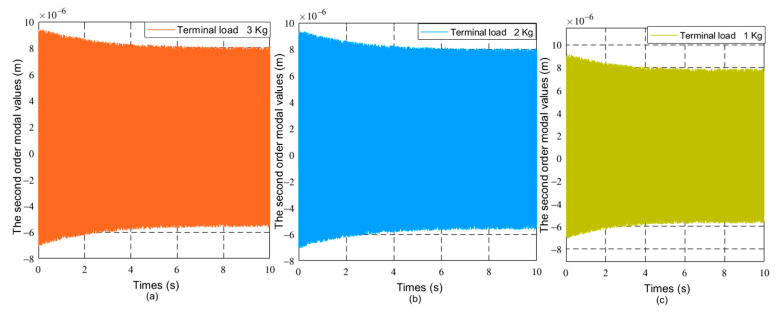
The second order modal changes of the manipulator under different loads, (**a**) 30 kg, (**b**) 20 Kg, (**c**) 10 Kg.

The driving moment of the joint is 80 N × m. The initial value of the generalized coordinates is ${\left[\begin{array}{cccc} \theta_{0} & {\dot{\theta }}_{0} & q & \dot{q} \end{array}\right]}^{T}={\left[\begin{array}{cccc} 0 & 0 & 0 & 0 \end{array}\right]}^{T}$. The dynamics of flexible robots with different loads and friction coefficients are studied. As the friction coefficient is 0, the terminal load mass is 1 Kg, 2 Kg, and 3 Kg respectively. As the terminal load mass is 1 Kg, the friction coefficient at the joint is 0.01.0.02 and 0.03, respectively. The joint angles and vibration modes of the flexible manipulator were compared under different parameters. Figure 4, Figure 5 and Figure 6 respectively show the joint angle and vibration mode diagrams under different loads. 

**Figure 7:**
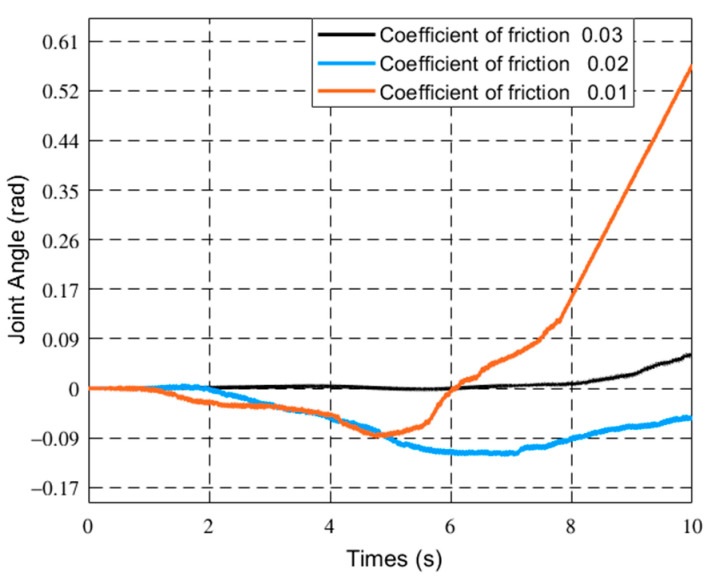
Joint angle changes of the manipulator under different friction coefficients.

**Figure 8:**
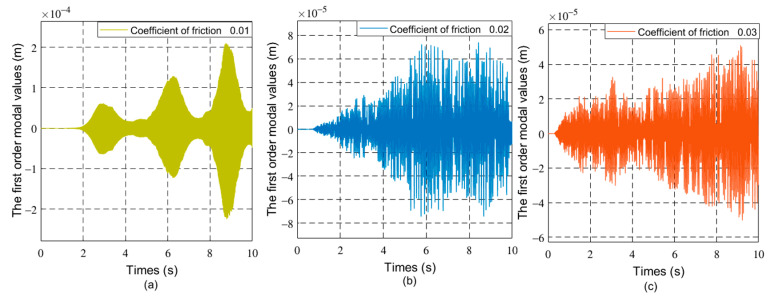
The first-order modal changes of the manipulator with different friction coefficients, (**a**) 0.01, (**b**) 0.02, (**c**) 0.03.

**Figure 9:**
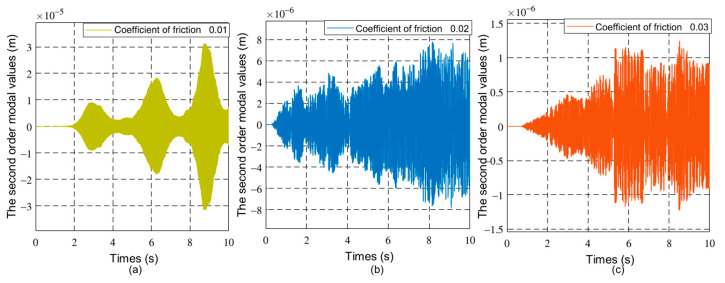
The second order modal changes of the manipulator with different friction coefficients, (**a**) 0.01, (**b**) 0.02, (**c**) 0.03.

Figure 7, Figure 8 and Figure 9 respectively show the joint angle and vibration modal diagrams with different friction coefficients.

In Figure 4, under the same torque, when the load increases, the joint angle of the flexible manipulator becomes smaller. Figure 5 and Figure 6 show that when the load increases, the frequency of the modal of the first order and the modal of the second order does not change, and the vibration amplitude increases. As shown in Figure 7, when the load mass is *M_L_* = 1 Kg and the friction coefficient is small (the friction coefficient is 0.01), the joint angle is mainly affected by the opposite force of elastic vibration and end-load. This also reflects the low friction coefficient of energy consumption. The variation trend of the joint angle under the influence of flexibility is basically the same as that of a rigid joint angle. In Figure 7, the joint angles of the flexible manipulator under the influence of different friction coefficients are further compared, clearly showing that the joint angle generally decreases with the increase of friction coefficient, and the vibration amplitude increases due to the presence of flexibility. Figure 8 and Figure 9 show that the frequency of the first and second vibration modes is reduced, and as the friction coefficient is 0.01, they present a periodized motion. The amplitude of the modal decreases with the increase of the friction coefficient. This is because friction consumes the energy of the driving force. The influence of the flexible characteristics on friction is taken into account and the original periodic changes are superimposed and offset. This makes the vibration an aperiodic motion.

### 5.2. Simulation of Control Method

**Figure 10:**
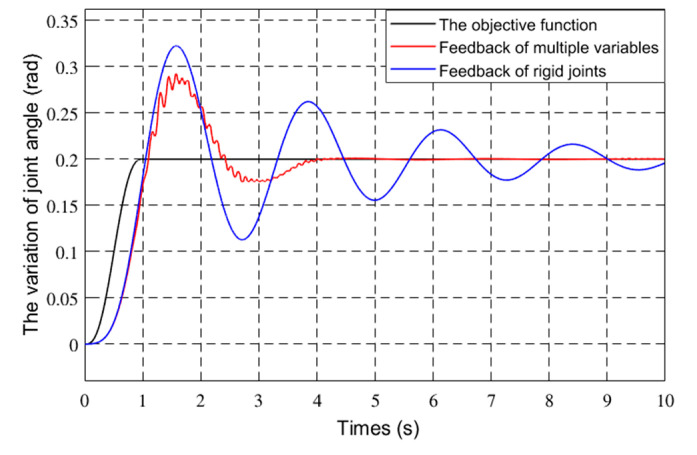
Joint angles variation of different controls.

**Figure 11:**
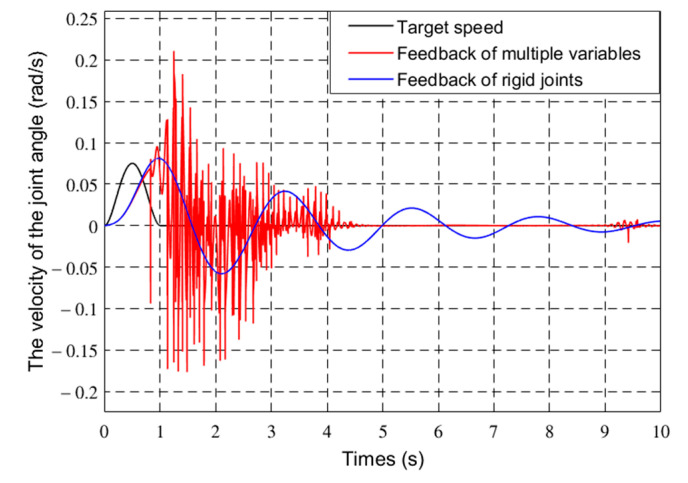
Joint angular velocity of different controls.

To verify the effectiveness of the control method, the Runge–Kutta method was used for simulation verification based on Matlab simulation platform. The mechanism parameters of the flexible manipulator are shown in Table 1. The terminal mass of the flexible manipulator is 2 Kg. The friction coefficient is 0.03. The desired objective function of the joint of the flexible manipulator is Equation (42). The generalized coordinate initial variable of the flexible manipulator is ${\left[\begin{array}{cccc} \theta_{0} & {\dot{\theta }}_{0} & q & \dot{q} \end{array}\right]}^{T}={\left[\begin{array}{cccc} 0 & 0 & 0 & 0 \end{array}\right]}^{T}$. The control parameters of the flexible manipulator are *k_v_* = 20, *k_p_* = 8, *K_qv_* = [300 300]*^T^*, *K_qp_* = [300 300]*^T^*. The joint angles and vibration modes of the flexible manipulator were compared with the rigid feedback and multiple feedback modes. Figure 10 and Figure 11 show the joint angle and velocity of the flexible robot.

**Figure 12:**
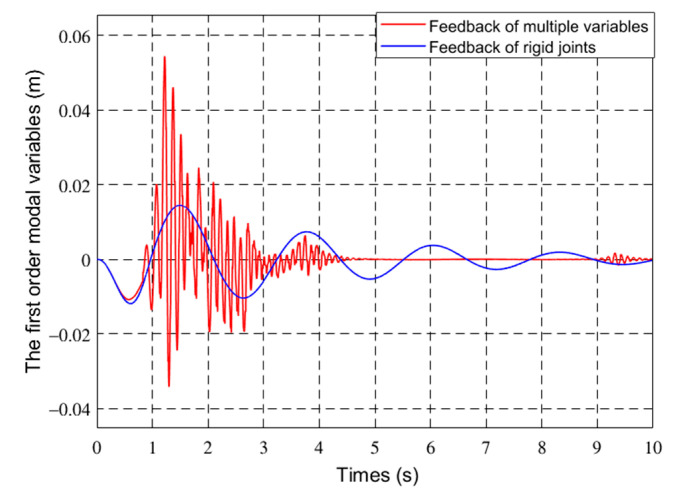
The first-order mode of different controls.

**Figure 13:**
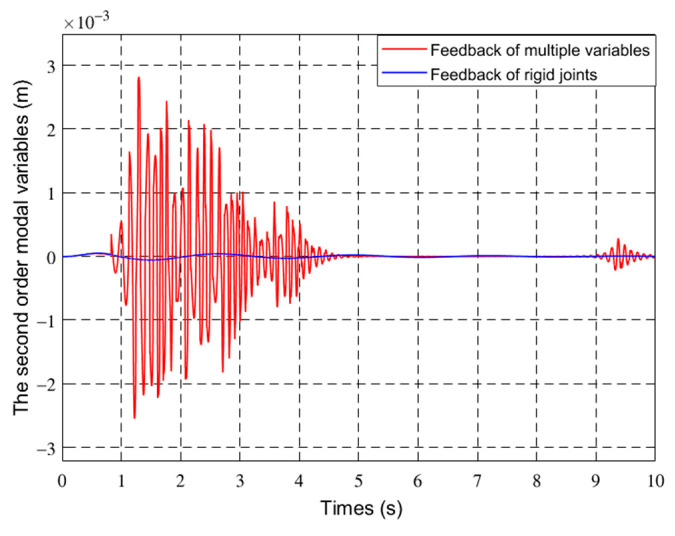
The second-order mode of different controls.

Figure 12 and Figure 13 show the first two modes of the flexible robot.

**Figure 14:**
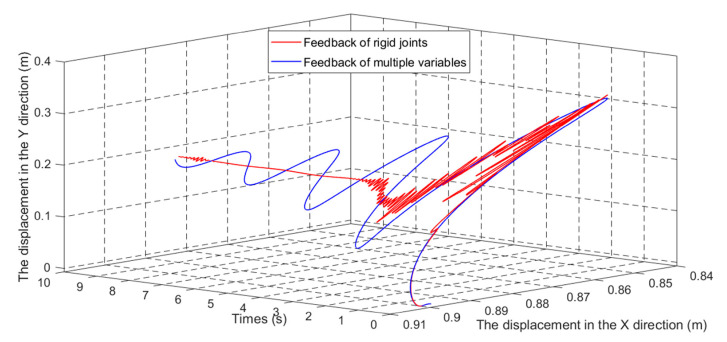
The terminal position change of flexible manipulator.

Figure 14 shows the end position of the flexible robot.

**Figure 15:**
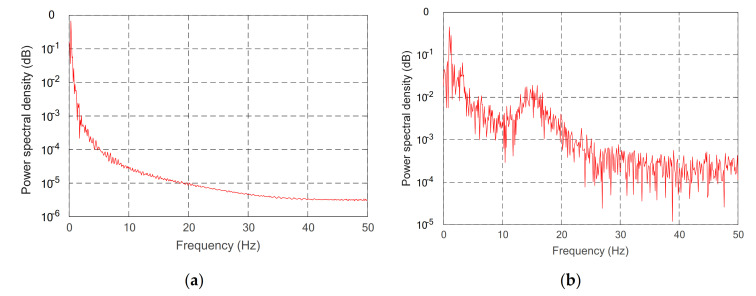
PSD of first-order modal variable of different controls, (**a**) controlled, (**b**) uncontrolled.

**Figure 16:**
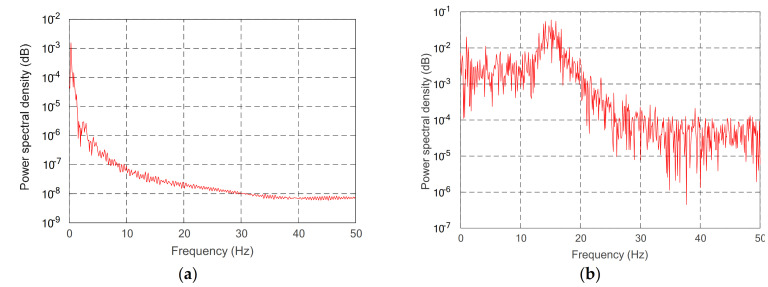
PSD of second-order modal variables of different controls, (**a**) controlled, (**b**) uncontrolled.

To further show the effectiveness of the control method, Power spectral density PSD of the uncontrolled and controlled model was further analyzed and studied. The second derivative and derivative of the given desired trajectory are substituted into Equation (32), and the uncontrolled moment is obtained. Using the periodic diagram power spectral density estimation method, the spectral density of the uncontrolled vibration mode variable is obtained. It is compared with the spectral density of vibration modal variables under the control model in Figure 15 and Figure 16.

(42) $$\theta_{d}=\left\{\begin{array}{c} 0.2\times \left[\left(6t^{5}/T^{5}\right)-\left(15t^{4}/T^{4}\right)+\left(10t^{3}/T^{3}\right)\right]\;\left(t<T,T=1\right)\\ \;0.2\;\left(t\geq T,T=1\right) \end{array}\right.$$

In Figure 11, Figure 12, Figure 13 and Figure 14, the red line vibration is mainly caused by the elastic body of the flexible manipulator. In this paper, due to the joint trajectory tracking, the driving torque is time-varying. The effect of time-varying friction force was further considered. This makes the driving torque coupling and time-varying. The vibration of the flexible manipulator is inevitable under the action of time-varying driving moment and flexible characteristics.

Through comparing the changes in the curve in Figure 10, the joint angle variation under multiple variable feedback control can not only track the target quickly but also suppress the vibration. Compared with the feedback of rigid joints, it can still achieve better feedback control. The further analysis of the velocity shows that the variation amplitude of joint angular velocity under multivariable feedback is large in Figure 11, but its change time is relatively short. Based on the comparison and analysis of the curve changes in Figure 12 and Figure 13, the amplitude of modal changes under multiple variable feedback control is large, but it can achieve fast tracking suppression in a very short time. Modal vibration is affected by the coupling of friction, gravity, and flexible characteristics. The modal amplitude is reduced under the feedback control of the rigid joints, but the vibration cannot be completely suppressed for a limited time. Similarly, through the analysis of the curve changes in Figure 14, it can be seen that the vibration at the end position under the multiple feedback controller can be quickly suppressed and the target can be well tracked after 4 s. According to the curve analysis in Figure 15a and Figure 16a, when the low frequency changes, the power density spectrum of the vibration modal displacement changes greatly. This is consistent with the low-frequency modal-based control research that is the main factor of vibration offset. When the frequency increases, the variation of the power density spectrum is small. This is consistent with the fact that the contribution of the higher frequency to the vibration modal offset is small. These trends prove the effectiveness of the control model. In Figure 15 and Figure 16, the PSD of modal variables under the uncontrolled (a) and controlled model (b) are compared. When the system frequency is close to the natural frequency of the system, the PSD reaches the peak point at the frequency. However, the whole flexible manipulator system has not only external damping, but also internal damping. The PSD will decrease gradually under the influence of mixed damping. Compared with the PSD in the non-control model, the PSD in the control model gradually decreases on the whole, and its amplitude fluctuates little. Therefore, the effectiveness of the control model is further proved by this conclusion.

## 6. Discussion

Considering the effects of a central rigid body and a curved flexible beam and loads, the dynamics of a complex coupled flexible manipulator were established. For further precise dynamics modeling, the effect of friction on the joints were considered. Compared with the previous friction model, the friction model not only considers the influence of gravity and rigid motion, but also considers the influence of flexible factors. Considering the friction effect, the dynamic model was further modified. The model is based on the combination of flexibility, terminal load, and improved friction effect. To control the flexible manipulator more conveniently and effectively, the coupling dynamics of the manipulator were decomposed. Flexible reaction targets were limited to a very small range and the flexible robot was controlled by a multi-loop feedback torque method. Compared with the previous feedforward control or complex control methods, it was simple and easy to implement. As the effective flexible control target approaches 0, the flexible manipulator can ensure that the control will be in the effective range.

Through the simulation analysis of the experiment, it can be seen that when the friction coefficient of the joint increases, the vibration of the mechanical arm presents an aperiodic vibration, and its vibration frequency decreases, and the amplitude of vibration significantly decreases. This is due to the great loss of driving energy caused by friction, and it is superimposed with the original vibration. As the terminal load increases in a certain range, the amplitude of vibration increases, and the vibration frequency almost has no obvious change. Under the multivariable feedback torque controller, the control method can effectively track the control trajectory, and the vibration of the internal flexible manipulator can be well suppressed within the effective time (*t* < 4 s). Compared with the rigid joint control, this control method has obvious good control effect.

## 7. Conclusions

In this paper, the center rigid body–flexible beam–load structure was selected as the research object. Considering the friction effect, the interaction effect between the flexible characteristics, and the terminal load, the dynamic model of the flexible robot with coupling characteristics was established. To realize track tracking and vibration suppression, the multiple closed-loop controllers were designed according to their rigid and flexible effects. The results show that the larger the terminal load is, the smaller the flexible vibration frequency is. but the larger the terminal load is, the larger the amplitude is. As the friction coefficient increases, the amplitude and vibration frequency decreases, and the vibration is aperiodic. Trajectory tracking and vibration suppression control were carried out for the nominal model with multiple feedback control. When the target of the flexible feedback approached the minimum value, and considering the interaction between the friction and the terminal load and the flexible vibration, the joint trajectory tracking and modal vibration suppression can be achieved within 4s. Finally, compared with the conventional PID rigid joint controller, it can be seen that the controller is effective and better.

In this paper, the dynamics of the manipulator with multiple factors such as flexibility, friction and terminal load were studied and analyzed. However, many factors such as joint flexibility, temperature effect, and air environment damping were not taken into account. The study of complex control methods under the influence of multiple factors needs further study. To meet the practical need, the dynamics of precision robots and more complex control research are the next steps. 

## Figures and Tables

**Table 1:** The parameters of the flexible robot mechanism.

Length *L* (m)	Density *ρ* (Kg/m)	Width *b* (m)	Height *h* (m)	Rotational Inertia *J* (Kg × m^2^)	Elasticity Modulus *EI* (N × m)	Tip Mass *M_Tip_* (Kg)
5	5.9292	0.06	0.04	5.12	6.21 × 10^7^	--

## Data Availability

No new data were created or analyzed in this study. Data sharing is not applicable to this article.

## Appendix A

As the modal interception order *N* of the flexible manipulator is generally 2, this paper takes *N* = 2 as an example for derivation. According to Equation (18), the following equations are further expressed.

$A_{i}=\int_{0}^{L}\phi_{i}^{2}\left(x\right)dx$, $B_{i}=\int_{0}^{L}\left(R+x\right)\phi_{i}\left(x\right)dx$, $E_{i}=\phi_{i}\left(L\right)$, $\eta_{i}=EI{\int_{0}^{L}\left(d^{2}\phi_{i}\left(x\right)/dx^{2}\right)}^{2}dx$ (*i* = 1,…,*N*)	

when *N* = 2.

$M=\left[\begin{array}{ccc} m_{11} & m_{12} & m_{13}\\ m_{21} & m_{22} & m_{32}\\ m_{31} & m_{32} & m_{33} \end{array}\right]$		$m_{11}=J+m_{p}L^{2}+\left(m/L\right)\left(A_{1}q_{1}^{2}+A_{2}q_{2}^{2}\right)+m_{p}\left(\left(E_{1}^{2}q_{1}^{2}+E_{2}^{2}q_{2}^{2}\right)\right)$	
$m_{22}=\left(m/L\right)A_{1}+m_{p}E_{1}^{2}$		$m_{33}=\left(m/L\right)A_{2}+m_{p}E_{2}^{2}$	$m_{12}=m_{21}=\left(m/L\right)B_{1}+m_{p}LE_{1}$
$m_{13}=m_{31}=\left(m/L\right)B_{2}+m_{p}LE_{2}$		$m_{23}=m_{32}=0$	
$C_{0}=2\dot{\theta }\left[\left(\left(m/L\right)A_{1}+m_{p}E_{1}^{2}\right){\dot{q}}_{1}+\left(\left(m/L\right)A_{2}+m_{p}E_{2}^{2}\right){\dot{q}}_{2}\right]$			$C_{1}=\left(\left(m/L\right)A_{1}+m_{p}E_{1}^{2}\right){\dot{\theta }}_{}^{2}q_{1}$
$C_{2}=\left(\left(m/L\right)A_{2}+m_{p}E_{2}^{2}\right){\dot{\theta }}_{}^{2}q_{2}$			$C={\left[\begin{array}{cccc} C_{0} & C_{1} & C_{2} & C_{3} \end{array}\right]}^{T}$
$G=mgL\cos\theta /2+m_{p}gL\cos\theta$			$K=diag\left(\left[\begin{array}{ccc} 0 & \eta_{1}^{2}q_{1} & \eta_{2}^{2}q_{2} \end{array}\right]\right)$
$A_{1}=\int_{0}^{L}\phi_{1}^{2}\left(x\right)dx$	$A_{2}=\int_{0}^{L}\phi_{2}^{2}\left(x\right)dx$	$B_{1}=\int_{0}^{L}\left(R+x\right)\phi_{1}\left(x\right)dx$	$B_{2}=\int_{0}^{L}\left(R+x\right)\phi_{2}\left(x\right)dx$
$E_{1}=\phi_{1}\left(L\right)$	$E_{2}=\phi_{2}\left(L\right)$	$\eta_{1}=EI{\int_{0}^{L}\left(d^{2}\phi_{1}\left(x\right)/dx^{2}\right)}^{2}dx$	$\eta_{2}=EI{\int_{0}^{L}\left(d^{2}\phi_{2}\left(x\right)/dx^{2}\right)}^{2}dx$
where *ϕ*(*x*) is a shape function, its specific equation is given as Equation (17).			
